# Nivolumab or Atezolizumab in the Second-Line Treatment of Advanced Non-Small Cell Lung Cancer? A Prognostic Index Based on Data from Daily Practice

**DOI:** 10.3390/jcm12062409

**Published:** 2023-03-21

**Authors:** Magdalena Knetki-Wróblewska, Sylwia Tabor, Aleksandra Piórek, Adam Płużański, Kinga Winiarczyk, Magdalena Zaborowska-Szmit, Katarzyna Zajda, Dariusz M. Kowalski, Maciej Krzakowski

**Affiliations:** Department of Lung Cancer and Chest Tumours, The Maria Skłodowska-Curie National Research Institute of Oncology, 02-781 Warsaw, Poland

**Keywords:** NSCLC, prognostic index, nivolumab, atezolizumab, immunotherapy

## Abstract

Background: The efficacy of nivolumab and atezolizumab in advanced pre-treated NSCLC was documented in prospective trials. We aim to confirm the benefits and indicate predictive factors for immunotherapy in daily practice. Methods: This study was a retrospective analysis. The median PFS and OS were estimated using the Kaplan-Meier method. The log-rank test was used for comparisons. Multivariate analyses were performed using the Cox regression method. Results: A total of 260 patients (ECOG 0-1) with advanced NSCLC (CS III-IV) were eligible to receive nivolumab or atezolizumab as second-line treatment. Median PFS and OS were three months (95% confidence interval [CI] 2.57–3.42) and 10 months (95% CI 8.03–11.96), respectively, for the overall population. The median OS for the atezolizumab arm was eight months (95% CI 5.89–10.1), while for the nivolumab group, it was 14 months (95% CI 10.02–17.97) (*p* = 0.018). The sum of all measurable changes >100.5 mm (*p* = 0.007; HR = 1.003, 95% CI 1.001–1.005), PLT > 281.5 G/l (*p* < 0.001; HR = 1.003, 95% CI 1.001–1.003) and bone metastases (*p* < 0.004; HR = 1.58, 95% CI 1.04–2.38) were independent negative prognostic factors for OS in multivariate analysis. Based on preliminary analyses, a prognostic index was constructed to obtain three prognostic groups. Median OS in the subgroups was 16 months (95% CI 13.3–18.7), seven months (95% CI 4.83–9.17) and four months (95% CI 2.88–5.13), respectively (*p* < 0.001). Conclusions: Nivolumab and atezolizumab provided clinical benefit in real life. Clinical and laboratory factors may help to identify subgroups likely to benefit. The use of prognostic indices may be valuable in clinical practice.

## 1. Introduction

Immune checkpoint inhibitors are an established standard of care for patients diagnosed with advanced non-small-cell lung cancer (NSCLC) after failure of chemotherapy. The CheckMate017, 057, and OAK trials demonstrated the superiority of nivolumab and atezolizumab over docetaxel in terms of OS [1,2,3,4]. Longer follow-up confirmed the value of both drugs. In a pooled efficacy analysis of nivolumab, the OS rates were 14% and 5% at four years and 13.4% and 2.6% at five years (HR 0.68, 95% CI 0.59–0.78). The greatest clinical benefit was observed in patients who achieved an objective response to treatment with nivolumab. In contrast, the proportions of patients who remained in follow-up after four years in the OAK trial were 15.5% and 8.7% for atezolizumab and docetaxel, respectively [5]. To date, there have been no prospective randomized trials comparing the efficacy and safety of the two drugs, and the number of retrospective analyses addressing this issue is limited [6,7,8]. At the same time, adequate qualification of patients for treatment remains an important issue in daily practice. The clinical profile of these patients differs from those evaluated in clinical trials. Moderate performance status, the presence of brain or liver metastases, significant localized lesions, and abnormalities in laboratory tests are the main challenges.

The aim of the study was to analyse the value of immunotherapy with nivolumab and atezolizumab in daily practice. The main objective was to identify the clinical and morphological factors that can define the profile of the patients who are most likely to benefit from the treatment in the long term.

## 2. Materials and Methods

A group of 260 patients qualified for nivolumab or atezolizumab treatment as part of daily practice at the Department of Lung and Chest Tumours of The Maria Skłodowska-Curie National Research Institute of Oncology in Poland between 2018 and 2021, and they were analysed. Eligibility criteria (in accordance with the local drug programme guidelines) included the diagnosis of stage III or IV NSCLC, one prior line of chemotherapy, good performance status (ECOG 0–1), measurable lesions detectable by computed tomography (CT), absence of clinically significant autoimmune disease, as well as molecular abnormalities of *EGFR* and *ALK* genes. Patients with brain metastases were eligible as long as they had received local treatment and were on a stable dose of corticosteroids within four weeks prior to starting immunotherapy. Previous use of PD-1/PD-L1 inhibitors was not permitted. Patients were assigned nivolumab or atezolizumab according to the physician’s discretion. Clinical and pathological data were obtained from available electronic medical records. Written informed consent was obtained from patients prior to initiation of immunotherapy. The local ethics committee approved the conduct of this analysis.

### 2.1. Efficacy Monitoring

A contrast-enhanced CT scan of the chest and upper abdomen (other areas are represented, as clinically indicated) was performed before starting immunotherapy. Treatment response was assessed using CT scans performed every three months, or more frequently if disease progression was clinically suspected. Treatment response was evaluated according to the Response Evaluation Criteria for Solid Tumours guidelines (RECIST 1.1). Treatment was continued until documented objective disease progression, unacceptable toxicity, or death for other reasons. Safety was assessed using the CTCAE, v. 5.0. Overall survival was defined as the time from the initiation of the second-line immunotherapy to death. Progression-free survival was defined as the time from the initiation of the immunotherapy to imaging progression, definite clinical progression, or death, whichever occurred first. Patients alive and without progression at the last observation were censored.

### 2.2. Statistical Analysis Methods

Survival analysis for PFS and OS was performed using the Kaplan-Meier method. For intergroup comparisons of baseline demographic and clinical variables, the chi-square test, median test, or Fisher-Freeman-Halton exact test (if the required assumptions for each test were met) was used. The logarithmic rank test was used for comparisons. Multivariate analyses were performed using Cox regression analysis. Survival parameters and the evaluation of the predictive and prognostic value of clinical and demographic parameters were performed based on the entire sample and in subgroups distinguished by the treatment modality. Analyses were performed using SPSS, v. 28.0.1.0, software.

## 3. Results

### 3.1. Characteristics of the Study Group

A total of 260 patients were eligible for the treatment; 134 patients received nivolumab, and 126 patients received atezolizumab. Table 1 summarises baseline demographic and clinical variables in the overall population and in subgroups according to the treatment modality. In the atezolizumab group, a higher proportion of patients had a diagnosis of lung adenocarcinoma, clinical stage IV, and these patients also reported presence of metastatic lesions in the brain. The differences were statistically significant.

### 3.2. Survival and Response to Treatment

**Figure 1:**
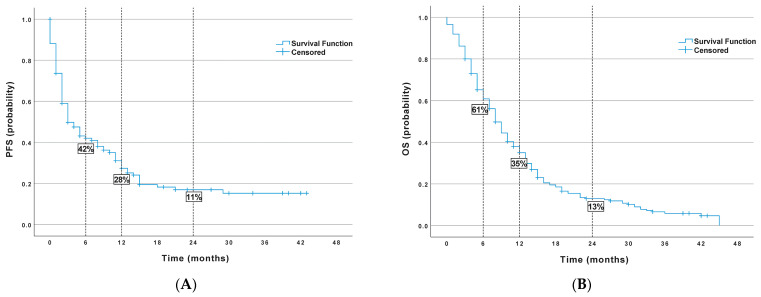
Probability of survival in the entire population (**A**) PFS (progression free survival), (**B**) OS (overall survival).

In the study group, an objective response to the treatment was recorded in 10.8% of the patients, and disease control was recorded in a total of 41.2% of the patients. In 40.4% of the patients, the best response to the treatment was disease progression. At the time of the analysis, 85 patients were still being followed (58 patients in the nivolumab group, 27 patients in the atezolizumab group), 35 patients were still receiving immunotherapy (26 patients were on nivolumab, and nine patients were on atezolizumab). Eighteen percent of the patients were lost to follow-up before the radiological assessment of the treatment response. The median PFS was three months, and the median OS was 10 months in the analysed group. (Figure 1.)

Differences in OS were observed between patients treated with nivolumab and atezolizumab (*p* = 0.018). Table 3 summarises the data on survival parameters.

#### 3.2.1. Univariate Analysis

**Figure 2:**
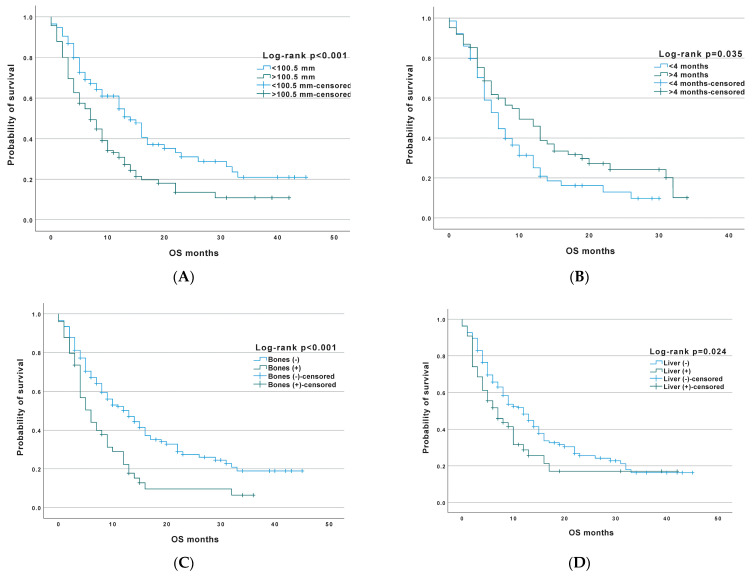
Overall survival among all patients in correlation to clinical factors: (**A**)—tumour diameter, (**B**)—time of response to chemotherapy, (**C**)—bone metastases, and (**D**)—liver metastases.

An analysis of the prognostic and predictive value of the selected factors that could be assessed prior to treatment initiation was performed. Table 4 summarises the results of the univariate analysis for the entire study group. Selected data are shown in Figure 2.

#### 3.2.2. Multivariate Analysis

The following variables were included in the model tested for both PFS and OS rates: type of the treatment regimen, age at start of treatment, and histopathological type. The latter two variables were included in the model because significant differences were observed between the groups according to treatment modality. In the model, the histopathological type is represented by two variables, adenocarcinoma (ADC; the presence of ADC vs. presence of other types) and squamous cell carcinoma (SCC; the presence of SCC vs. presence of other types). The tested model was not statistically significant for PFS (χ^2^ (df = 4) = 3.57, *p* = 0.467), and no statistically significant differences were observed for OS (χ^2^ (df = 4) = 6.359, *p* = 0.174) Results are summarised the Table 5. In conclusion, the type of immunotherapy used had no effect on the survival parameters when the concomitant variables (age and histopathological type) were taken into account.

Neutrophil levels and duration of response to chemotherapy were found to be the most significant predictors for PFS (the tested model was statistically significant (χ^2^ (df = 6) = 50.109, *p* < 0.001)), while, for OS, the most significant predictors included total measurable lesions, presence of bone metastatic lesions, and elevated platelet count (the tested model was statistically significant (χ^2^ (df = 9) = 72.163, *p* < 0.001)). The data are presented in Table 6.

#### 3.2.3. Prognostic Index

The starting point for the development of the prognostic index was a preliminary model to analyse the relevance of clinical and demographic parameters for the OS in the univariate analysis. Based on this, a final model was constructed, which included variables that were significant in the preliminary model: the presence of bone metastases, elevated platelet levels, and the dimension of measurable lesions. The model was tested in a sample consisting of randomly selected subjects, representing 60% of the total sample, and then these subjects were validated in a group, representing the remaining 40%. In the first group, the model was statistically significant for χ^2^ (df = 3) = 91.551, *p* < 0.001. A similar result was obtained in the validation group with χ^2^ (df = 3) = 47.706, *p* < 0.001. Using the estimated regression coefficients (B), a prognostic index was calculated using the following formula: PI = 0.002 × (platelets) + 0.003 × (bones) − 0.725 × (sum of all measurable changes)

The next step was to construct a simplified prognostic index. For this purpose, a Cox regression analysis was performed, and the variables listed, treated as categorical ones, were introduced as predictors. The k-means method was used to separate two groups for plaque levels and tumour totals. Table 7 shows the estimated regression coefficients for each variable in the model and the scores assigned to them.

**Figure 3:**
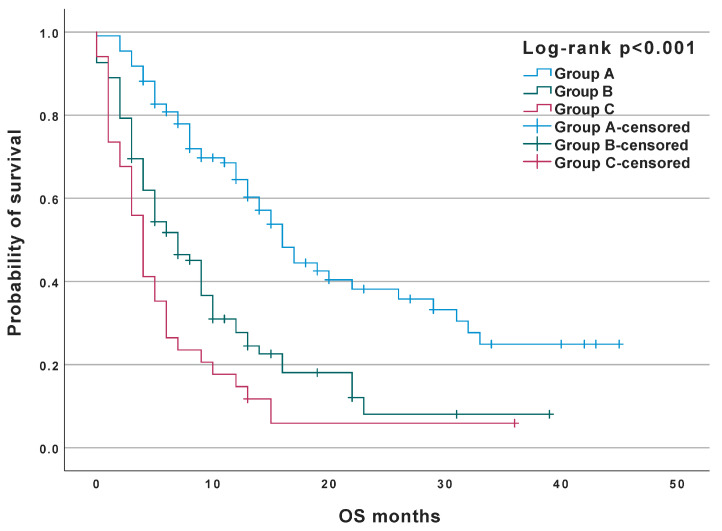
Probability of survival in the prognostic groups.

Patients with a similar index score were grouped into three prognostic groups: Favourable—index value 0, Intermediate—index values 2–3, and Poor—index values 5–8. Table 8 presents the characteristics of the three groups in relation to the variables comprising the prognostic index and the median OS. The Kaplan-Meier curves for the three prognostic groups are shown in Figure 3.

## 4. Discussion

The population of patients eligible for immunotherapy after failure of platinum-based chemotherapy is heterogeneous. Disease progression is often associated with a deterioration of the general condition in many cases. In addition, liver or central nervous system metastases may affect the outcome of the treatment [1,2,3]. Immunotherapy is one of the possible therapeutic options, along with the chemotherapy combined with anti-angiogenic treatment, palliative radiotherapy, or systemic treatment used in clinical trials. The decision on the choice of the therapy is primarily based on an assessment of prognostic factors, the most important of which is the performance status of the patient. Our results indicate other key prognostic factors.

### 4.1. Tumour Size

The sum of measurable changes may have prognostic significance, although there are no consistent data on that for immune checkpoint inhibitors. In the presented patient group, the median measurable lesions size was 100 mm, and significantly shorter PFS and OS were observed in the group of patients whose total lesions size exceeded this value. Similar observations were published by Katsaruda et al. In an analysis of 58 patients receiving immunotherapy, it was observed that patients with total tumour size greater than 101 mm had significantly worse PFS (2.07 months versus 6.39 months; *p* = 0.044) and OS (5.85 months versus 22.28 months; *p* < 0.01) than patients with smaller lesions [9]. In a study by Uehara et al. of 191 patients receiving immunotherapy alone or in combination with chemotherapy, a multivariate analysis showed that lesion size greater than 50 mm was an independent negative predictor of PFS in the monotherapy group. This association was not observed in the group of patients treated with combined chemotherapy and immunotherapy [10]. Although, in some research articles, the size of the tumour is a factor that correlates with response to immunotherapy, and there are also observations that do not support this association. In response to the immunotherapy, the sum of measurable changes may not be important per se, but rather, this may be the result of the disease dynamics, which often correlate with parameters of tumour aggressiveness, such as LDH [11].

### 4.2. Liver Metastases

The liver is one of the most common organ sites for metastases from malignant tumours, including lung, gastrointestinal, and breast cancers. In lung cancer, the incidence of distant liver metastases varies between 3 and 21%, depending on the subtype. The presence of distant liver metastases is a known negative prognostic factor in both NSCLC and small-cell lung cancer (SCLC) [12,13]. The mechanism of their formation is related to a number of molecular factors, such as the expression of specific genes or regulation at the microRNA (miRNA) level [14]. Based on the available results of the randomised clinical trials, it also appears that the presence of liver cancer metastases correlates with a poorer response and shorter overall survival in patients treated with immunotherapy. In preclinical models, it has been shown that, by regulating the tumour microenvironment, they affect the reduction of CD8+ lymphocytes in the systemic circulation by activating their apoptosis, which occurs in the liver, directly leading to a reduced benefit of immunotherapy [14].

The results of a meta-analysis evaluating data from immunotherapy trials in patients with liver metastases confirmed the lack of effect of PD-1/PD-L1 inhibitor monotherapy on PFS prolongation compared to chemotherapy [15]. In a pooled analysis of the CheckMate 017 and CheckMate 057 study trials at three years of follow-up, a median OS in the nivolumab arm was demonstrated to be longer by 2.2 months when compared to the chemotherapy arm in the subgroup of patients with liver metastases diagnosed at baseline. However, the median OS in this group of patients was only 6.8 months (for nivolumab) [16]. Similar analyses were not performed in the atezolizumab trials [4,17,18]. Data from patients with treated baseline liver metastases, presented in the phase III IMpower150 trial, confirmed the negative impact on OS [19]. The median OS for this subgroup was 13.3 months in the ABCP (atezolizumab, bevacizumab, carboplatin, paclitaxel) experimental arm and 9.4 months in the BCP (bevacizumab, carboplatin, paclitaxel) control arm. Again, the median OS obtained was shorter than that seen in the overall patient population in the trial [19].

The data presented here confirm the negative impact of distant liver metastases from NSCLC on the survival time in patients treated with PD-1/PD-L1 checkpoint inhibitors.

### 4.3. Body Mass Index

Our study suggests that the nutritional status of patients is important for the efficacy of immunotherapy. Based on the results of preclinical studies, it should be noted that white adipose tissue, which is crucial for the process of weight gain, is also involved in the induction and coordination of host defence mechanisms, being a source of cytokines and chemokines [20]. Adipose tissue modulates the balance of Th1/Th2 lymphocytes, reduces the activation of regulatory T cells by adiponectin, increases the number of pro-inflammatory macrophages, and enhances the pro-inflammatory process through the CD40 pathway [21].

Furthermore, it has been shown in preclinical models that white adipose tissue may also play a role in immune homeostasis [22]. Mouse white adipose tissue was found to accumulate pathogen-specific memory T cells after bacterial infection. These data support the hypothesis that adipose tissue may be a reservoir of tissue-specific memory T cells that can be rapidly reactivated in the face of external stimuli. The question remains whether adipose-specific T cells can be reactivated equally rapidly against cancer-specific antigens. Analyses of the effect of high BMI and survival parameters, which have been performed in populations of patients diagnosed with melanoma, lung cancer, and other cancers, indicate that patients with a higher BMI benefit more clinically from immunotherapy [23]. A multicentre retrospective study of 1070 patients diagnosed with non-small-cell lung cancer, melanoma, and renal cell carcinoma treated with pembrolizumab, nivolumab, and atezolizumab evaluated the relationship between BMI, OS, and PFS [24]. The median PFS for patients with BMI >30 was shown to be 12.9 months compared to 1.9 months for underweight patients (BMI < 18.5) and 4.4 months for normal weight patients (BMI >18.5 <24.9). Median OS was not reached for obese patients, while it was 8.0 months for patients of normal weight [24].

### 4.4. Duration of Response to Prior Chemotherapy

In the analysed patients’ group, the median time from the end of chemotherapy to the disease progression was four months. Patients in whom the duration of response was shorter than median, and the median were less likely to benefit from second-line immunotherapy. This observation is consistent with data from other authors. Therefore, the length of the observation period until disease progression may be a subject of debate [25]. In the CheckMate 057 study, an improvement in OS was observed in patients who had ended chemotherapy more than six months before (HR 0.46, 95%CI 0.27–0.79) [1]. However, in the CheckMate017 study, no such association was observed in patients who had stopped chemotherapy more than six months before randomisation, and there was no significant benefit of nivolumab over docetaxel in terms of OS (HR 0.64, 95% CI 0.37–1.13) [2].

### 4.5. Inflammatory Blood Biomarkers

The LIPI score, an index that combines dNLR (=neutrophil count/white blood cell count—neutrophil count) and blood lactate dehydrogenase (LDH) levels, has been developed to predict the effectiveness of immunotherapy [26]. In a first available study on the subject, LIPI was divided into three categories: good (dNLR 3 and LDH upper limit of normal), intermediate (dNLR > 3 or LDH > GGN) and poor (dNLR > 3 and LDH > upper limit of normal) [27]. Indeed, patients with poor LIPI were shown to have the worst prognosis, i.e., the shortest PFS and OS. Furthermore, intermediate and poor LIPI independently correlated with progression detected at the first radiological assessment: OR = 2.20 (*p* = 0.005) for intermediate, and OR = 3.04 (*p* = 0.003) for poor LIPI, respectively. All the differences were significant only in patients treated with immunotherapy; no differences were found in chemotherapy-treated patients. In another study, good LIPI was shown to be associated with longer OS when compared to poor LIPI. Furthermore, as in the previous study, patients with NSCLC and poor LIPI did not benefit from immunotherapy when compared to chemotherapy (no advantage of immunotherapy over chemotherapy) [28]. For the first time, this study also demonstrated the usefulness of LIPI to predict survival not only during immunotherapy, but also during targeted therapy and chemotherapy. A pooled analysis of results from several clinical trials comparing the efficacy of atezolizumab with docetaxel (BIRCH, FIR, OAK, POPLAR) showed that LIPI groups were significantly associated with PFS and OS; furthermore, the superiority of atezolizumab over docetaxel in terms of PFS and OS was only observed for good and intermediate LIPI groups [29]. Patients treated with nivolumab in second-line NSCLC also showed a correlation between LIPI groups and OS [30]. The poor LIPI group had significantly shorter OS in both univariate (HR = 3.12; *p* < 0.0001) and multivariate analysis (HR = 3.67; *p* < 0.0001). Such an association was not seen between LIPI and PFS in multivariate analysis. However, it should be noted that poor LIPI correlated with lower odds of achieving disease control (OR = 0.44; *p* = 0.005). An available meta-analysis of four clinical trials involving a total of 7373 patients with NSCLC receiving immunotherapy, targeted therapies, or chemotherapy demonstrated that LIPI groups can be effectively used to determine prognosis [31]. The LIPI groups predicted OS both in patients treated with immunotherapy and in those treated with targeted therapies or chemotherapy. Only in patients with squamous cell carcinoma treated with chemotherapy was there no effect of LIPI on OS. In daily practice (real-world data), LIPI groups proved to have prognostic significance during immunotherapy treatment, not only in patients with NSCLC, but also in patients with other cancers, such as RCC and melanoma [32].

Neutrophils and platelets play an important role in tumour development and progression, as well as in metastasis—directly affecting tumour cells or indirectly affecting other components of the tumour microenvironment [33,34]. Some data suggest that platelets can influence tumour cells, which may lead to the promotion of an unfavourable phenotype in the form of increased proliferation [35]. Furthermore, the PD-L1 protein has been shown to be transferred from tumour cells to platelets [36]. In contrast, neutrophils are one of the most prevalent immune cells in NSCLC, accounting for almost 20% of all immune cells, and they are potentially immunosuppressive in this disease [37]. The results of several meta-analyses suggest a predictive role of NLR for immunotherapy [38,39,40,41,42]. A recent article included a total of 23 trials with a total of 2068 patients with NSCLC [43]. Six trials used first-line immunotherapy, and the remainder used second- or subsequent-line therapy. Most of the patients received nivolumab as immunotherapy. Twenty reports involving 1629 patients were used to analyse the correlation of NLR before treatment and OS. The results of the study once again showed that a higher NLR was associated with worse OS.

The results of the meta-analysis demonstrated the prognostic value of PLR in lung cancer patients receiving immunotherapy. Based on 14 retrospective studies involving 1761 patients, elevated pre-treatment PLR was found to be significantly correlated with worse OS in these patients [44]. Similar results were found in two previous meta-analyses [41]. The prognostic value of platelet count in relation to OS in patients with lung cancer was analysed in a systematic review of 39 studies with 16,570 patients. A negative impact on OS was indicated in multivariate analysis. In our study, platelet count >281.5 G/l was a negative prognostic factor in both univariate and multivariate analyses. We found no previously published data on this abnormality in patients treated for NSCLC with checkpoint inhibitors.

### 4.6. Bone Metastases

Bone metastases are a significant clinical problem in patients diagnosed with NSCLC, being observed in more than 30% of patients [45]. The occurrence of skeletal adverse events, including pathological fractures or spinal cord compression, may correlate with poorer quality of life and shorter overall survival, regardless of the type of systemic treatment used [45]. In our analysis, the presence of metastatic bone lesions was an independent negative prognostic factor and was also considered as one of the variables of the prognostic index we developed. Other authors confirm these observations in relation to the population of patients eligible for immunotherapy. Landi et al. published the results of an analysis of a large cohort of patients treated with nivolumab in the Italian Drug Access Programme [46]. The population consisted of cohort A, which included 1588 patients with non-squamous NSCLC, including 39% of patients with bone metastases, and cohort B accounted for 371 patients with squamous histology, including 32% of patients with bone metastases. In both cohorts, bone metastases were found to be a significant independent negative prognostic factor with respect to OS with HR 1.50 (95% CI 1.30–1.73; *p* < 0.0001) for cohort A and HR 1.78 (95%CI 1.37–2.31; *p* < 0.0001) for cohort B [46]. The significant shortening of OS in patients with bone metastatic lesions has also been confirmed by other authors publishing data from analyses based on large groups of patients treated in daily practice [45,47,48]. The evaluation of the efficacy and safety of immunotherapy combined with denosumab, used to prevent of skeletal adverse events in patients with bone metastases, is still under discussion [49,50].

### 4.7. Nivolumab or Atezolizumab?

In the study presented here, the analysis evaluating the effect on OS showed some advantages for nivolumab (*p* = 0.018). However, further analyses and a multivariate model showed no significant differences. Differences in the distribution of characteristics between subgroups (a higher proportion of patients with CNS metastases and a higher proportion of patients with clinical stage IV in the atezolizumab-treated group) should be highlighted. The retrospective nature of our analysis further limits the value of this observation. Other authors have also reported a greater prognostic significance of the clinical factors analysed than of the drug used [5,6].

### 4.8. Prognostic Indices

Based on our analyses, we developed a prognostic index that takes into account three clinical variables: the presence of bone metastases, large tumour size, and an elevated platelet count. This allowed us to identify groups with different prognoses. The index will be validated in subsequent cohorts of patients. Other authors have published prognostic indices, which were based on additional parameters, such as albumin and bilirubin levels, histological type, response status to previous chemotherapy, presence of liver metastases, LDH, NLR, performance status, etc. [51,52,53,54,55]. We emphasize the value of using prognostic indices based on clinical parameters that can be easily applied before qualifying patients for the treatment. For example, the EPSILoN index, based on smoking, ECOG, liver metastases, LDH, and NLR, allowed the identification of three groups of patients with significantly different prognoses: median OS of 24.5, 8.9, and 3.4 months, respectively (HR 2.40, *p* < 0.001) [54,55]. Given the limited efficacy of immunotherapy in second-line treatment of advanced NSCLC and the high proportion of patients with confirmed disease progression in the first weeks of therapy, the implementation of such tools may realistically optimise the qualification of patients for treatment.

## 5. Conclusions

Our study includes patients who were eligible for immunotherapy in routine practice. The retrospective nature of this single-centre analysis and the relatively small number of patients are limitations of the study. Nivolumab and atezolizumab produced similar clinical benefits, but despite the good performance status of the patients (ECOG 0–1), the treatment outcomes in the analysed cohort were worse than in clinical trials. Clinical and laboratory factors may help to identify subgroups likely to respond to the treatment. The most important positive prognostic factors appear to be small tumour diameter, absence of liver and bone metastases, long duration of response to previous chemotherapy, and some laboratory parameters. The use of prognostic indices may be valuable in the assessment of overall survival. The prognostic index we have developed is based on simple clinical variables and indicates significant differences in the likelihood of long-term clinical benefit.

## Figures and Tables

**Table 1:** Baseline demographic and clinical characteristics by type of the treatment.

	Total (n = 260)	Nivolumab (n = 134)	Atezolizumab (n = 126)	*p*-Values
Age				
Median (range)	67 (37–91)	68 (39–91)	66 (37–83)	0.025
Gender				
Female	111 (42.7%)	54 (40.3%)	57 (45.2%)	0.421
Male	149 (57.3%)	80 (59.7%)	69 (54.8%)	
Histology				
ADC	137 (52.7%)	29 (21.6%)	108 (85.7%)	<0.001
SCC	102 (39.2%)	93 (69.4%)	9 (7.1%)	
LCC	2 (0.8%)	2 (1.5%)	0 (0%)	
NOS	9 (3.5%)	4 (3%)	5 (4%)	
LCNEC	4 (1.5%)	1 (0.7%)	3 (2.4%)	
Poorly differentiated	6 (2.3%)	5 (3.7%)	1 (0.8%)	
Smoking (pack years)				
Median (range)	30 (0–80)	30 (0–80)	30 (0–55)	0.709
Clinical stage				
III	64 (24.6%)	48 (35.8%)	16 (12.7%)	<0.001
IV	196 (75.4%)	86 (64.2%)	110 (87.3%)	
Metastases				
Brain	33 (9.2%)	14 (10.7%)	19 (16.5%)	<0.001 ^e^
Lungs	137 (38.3%)	85 (64.9%)	52 (45.2%)	
Bones	49 (13.7%)	23 (17.6%)	26 (22.6%)	
Liver	54 (15.1%)	31 (23.7%)	23 (20%)	
Adrenal glands	46 (12.8%)	19 (14.5%)	27 (23.5%)	
Other	39 (10.9%)	9 (6.9%)	30 (26.1%)	
ECOG				
0	10 (3.8%)	6 (4.5%)	4 (3.2%)	0.821
1	246 (94.6%)	126 (94%)	120 (96%)	
2	3 (1.2%)	2 (1.5%)	1 (0.8%)	
Sum of all measurable lesions (in mm)				
Median (range)	100.5 (10–454)	90.5 (10–454)	110 (10–390)	0.291
Duration of response to chemotherapy				
Median months (95% CI)	4.0 (3.0 to 5.0)	4.0 (3.0 to 5.0)	4.0 (3.0 to 5.0)	0.345
Time of observation				
Median months (95% CI)	19.0 (15.0 to 21.0)	13.0 (12.0 to 13.0)	24.0 (22.0 to 26.0)	<0.001

ADC—adenocarcinoma, SCC—squamous carcinoma, LCC—large cell carcinoma, NOS—non-otherwise specified, LCNEC—large cell neuroendocrine carcinoma, ECOG—Eastern Cooperative Oncology Group, Table 2 summarises the response to prior chemotherapy in the study group and in both subgroups distinguished by the treatment modality. Differences between subgroups were statistically significant (*p* < 0.001).

**Table 2:** Response to previous chemotherapy.

	Total (n = 248)	Nivolumab (n = 125)	Atezolizumab (n = 123)	*p*-Value
Best response				<0.001
CR	16 (6.5%)	11 (8.8%)	5 (4.1%)	
PR	45 (18.1%)	11 (8.8%)	34 (27.6%)	
SD	103 (41.5%)	51 (40.8%)	52 (42.3%)	
PD	84 (33.9%)	52 (41.6%)	32 (26%)	

CR—complete response, PR—partial response, SD—stable disease, PD—progressive disease.

**Table 3:** Treatment outcomes in the general population and subgroups analysed (nivolumab vs. atezolizumab).

	Total (n = 260)	Nivolumab (n = 134)	Atezolizumab (n = 126)	*p*-Value
Best response				
CR	1.2%	1.4%	0.8%	0.592
PR	9.6%	5.4%	8.7%	
SD	30.4%	30%	30.1%	
PD	40.4%	35.1%	46%	
ORR	10.8%	6.8%	9.5%	0.36
DCR	41.2%	36.8%	39.5%	0.222
Lack of data	18.4%	22.3%	14.4%	
Progression-free survival				
Median months (95% CI)	3 (2.57–3.42)	3 (2.27–3.72)	3 (2.49–3.50)	0.175
Overall survival				
Median months (95% CI)	10 (8.03–10.9)	14 (10.02–7.97)	8 (5.89–10.1)	0.018

CR—complete response, PR—partial response, SD—stable disease, PD—progressive disease, ORR- objective response rate, DCR—disease control rate.

**Table 4:** Univariate analysis for all patients.

Characteristic	PFS			OS		
	All pts			All pts		
	HR	95%CI	*p*-Value	HR	95%CI	*p*-Value
Age (< vs. >67 years)	0.97	0.74–1.26	0.796	1.07	0.8–1.44	0.637
Squamous vs. nonsquamous	0.83	0.63–1.09	0.177	0.7	0.51–0.96	0.028
Female vs. Male	1.21	0.92–1.58	0.17	1.04	0.77–1.4	0.81
SML > mediane	1.46	1.11–1.93	0.008	1.79	1.3–2.47	<0.001
Liver metastases	1.42	1.03–1.96	0.034	1.49	1.04–2.14	0.03
Bone metastases	1.27	0.92–1.77	0.152	1.91	1.35–2.71	<0.001
Time of response to chemotherapy < 4 months *	1.7	1.29–2.23	<0.001	1.83	1.34–2.35	<0.001
BMI > 25.63 **	0.83	0.64–1.07	0.154	0.74	0.55–1	<0.047
NEU > 5.39 **	1.44	1.11–1.87	0.007	1.43	1.06–1.93	0.018
PLT > 281 **	1.26	0.97–1.64	0.085	1.48	1.1–2	0.01
Lymphocyte > 1.44 **	0.67	0.51–0.87	0.002	0.57	0.42–0.77	<0.001
NLR > 3.68 **	1.79	1.38–2.34	<0.001	2.08	1.54–2.82	<0.001
PLR > 182.9 **	1.76	1.35–2.29	<0.001	2.23	1.64–3.04	<0.001
LIPI 2 vs. 0	1.42	0.92–2.2	0.11	1.69	1.03–2.78	0.039
Albumine < 38.8	1.57	1.06–2.33	0.023	2.16	1.39–3.37	<0.001

SML—sum of measurable lesions (for the entire population the median was 100.5 mm, for nivolumab it was 95 mm, and for atezolizumab it was 110 mm), * median time of response to chemotherapy was four months, ** median of the analysed value; BMI—body mass index, NEU—neutrophils, PLT—platelets, NLR—neutrophiles/lymphocytes ratio, PLR—platelets/lymphocytes ratio, LIPI—Lung Immune Prognostic Index, PFS—progression free survival, OS-overall survival.

**Table 5:** Statistics for the variables in the tested model.

	PFS				OS			
	HR	SE	Wald	*p*-Value	HR	SE	Wald	*p*-Value
Type of treatment	0.206	0.19	1.174	0.279	0.235	0.211	1.237	0.266
Age	0.002	0.008	0.069	0.793	0.002	0.01	0.037	0.847
ADC	0.243	0.255	0.909	0.34	0.143	0.292	0.241	0.624
SCC	0.249	0.267	0.872	0.35	0.320	0.313	1.045	0.307

PFS—progression free survival, OS—overall survival, ADC—adenocarcinoma, SCC—squamous carcinoma, HR—hazard ratio, SE—standard error.

**Table 6:** Statistics for the variables in the tested model: multivariate analysis.

	PFS			OS		
	*p*-Value	HR	95% CI	*p*-Value	HR	95% CI
Sum of all measurable lesions (>100.5 mm)	0.210	1.001	0.99–1.003	0.007	1.003	1.001–1.005
Liver metastases	0.141	0.754	0.519–1.098	0.309	0.803	0.527–1.225
Time of duration of response to chemotherapy < 4 months	0.025	0.969	0.94–0.99	0.068	0.970	0.939–1.002
Neutrophils >5.39 × 10^3^/μl	0.001	1.046	1.017–1.075	0.157	1.024	0.991–1.059
Lymphocytes <1.44 × 10^3^/μl	0.812	0.989	0.905–1.081	0.690	0.979	0.883–1.085
Platelets > 281 G/L	-	-	-	<0.001	1.003	1.001–1.004

PFS—progression free survival, OS—overall survival.

**Table 7:** Prognostic index.

	Coefficient	Points
Sum of all measurable changes (<163 mm vs. >163 mm)		
<163 mm	0	0
>163 mm	0.62	2
Bones		
No	0	0
Yes	0.734	3
Platelets		
<356	0	0
>356	0.706	3

**Table 8:** Clinical characteristics of the prognostic groups.

	Favourable—Risk Group A (0 Points)	Intermediate—Risk Group B (2–3 Points)	Poor—Risk Group C (5–8 Points)
Sum of all measurable changes			
Mean (SD)	77.39 (39.75)	137.44 (80.63)	193.85 (92.22)
Bones metastases (%)			
No	100%	74.4%	35.3%
Yes	0%	25.6%	64.7%
Platelets			
Mean (SD)	253 (54.76)	335.76 (130.05)	395.23 (134.8)
Overall survival			
Median months (95% CI)	16 (13.3–18.7)	7 (4.83–9.17)	4 (2.88–5.13)

## Data Availability

The data that support the findings of this study are available from the corresponding author upon reasonable request.
